# Supplementary material for: Variance heterogeneity genome‐wide mapping for cadmium in bread wheat reveals novel genomic loci and epistatic interactions
Source: Plant Genome. 2020 Apr 11;13(1):e20011. doi: 10.1002/tpg2.20011 (PMC12807127; doi:10.1002/tpg2.20011)
Supplement: Supplementary file 1 [file TPG2-13-e20011-s001.docx]

**Supplemental Material**

**Tables**

| **Table S1**: Single nucleotide polymorphism markers associated with variance heterogeneity of cadmium concentration in the hard-red winter wheat association panel. | | | | | | | | | | |
| --- | --- | --- | --- | --- | --- | --- | --- | --- | --- | --- |
| SNP: | Chr^1^ | P-values | | Alleles | Pos(bp)^2^ | MJF^3^ | MAF^4^ | N^5^ | AA^6^ | BB^6^ |
|  |  | DGLM | HGLM |  |  |  |  |  |  |  |
| IWA4096 | 2B | 6.50E-07 | 4.80E-08 | G/C | 696677568 | 0.89 | 0.10 | 299 | 268 | 31 |
| IWA4097 | 2B | 3.46E-06 | 4.79E-07 | T/C | 696678792 | 0.91 | 0.08 | 299 | 274 | 25 |
| IWA4095 | 2B | 3.71E-06 | 4.89E-07 | A/G | 696677327 | 0.90 | 0.09 | 299 | 270 | 29 |
| Excalibur_c36280_764 | 2B | 2.49E-06 | 4.83E-07 | T/G | 697512104 | 0.89 | 0.10 | 299 | 267 | 32 |
| IWA2379 | 2B | 2.69E-06 | 4.83E-07 | T/C | 701097263 | 0.87 | 0.12 | 299 | 261 | 38 |
| BS00046164_51 | 2B | 3.13E-06 | 4.22E-07 | A/C | 697510373 | 0.90 | 0.09 | 299 | 270 | 29 |
| IWA2702 | 2B | 2.92E-06 | 4.59E-07 | T/C | 700456554 | 0.89 | 0.10 | 299 | 267 | 32 |
| IAAV6424 | 2B | 3.67E-07 | 5.00E-07 | T/C | 691780716 | 0.87 | 0.12 | 299 | 261 | 38 |
| IAAV3067 | 2B | 1.36E-06 | 5.56E-06 | C/G | 699173434 | 0.90 | 0.09 | 299 | 271 | 28 |
| Kukri_c834_259 | 2B | 1.20E-06 | 4.89E-06 | T/C | 698968658 | 0.91 | 0.08 | 299 | 273 | 26 |
| IAAV2917 | 2B | 9.87E-06 | 5.64E-06 | A/G | 697511682 | 0.88 | 0.11 | 299 | 266 | 33 |
| BobWhite_c2521_117 | 2B | 1.31E-06 | 3.92E-06 | A/G | 699106309 | 0.87 | 0.12 | 299 | 262 | 37 |
| Ex_c16948_754 | 2B | 1.80E-06 | 4.15E-06 | A/G | 699827018 | 0.91 | 0.08 | 299 | 274 | 25 |
| IWA4909 | 2B | 1.80E-06 | 4.15E-06 | A/G | 700207481 | 0.91 | 0.08 | 299 | 274 | 25 |
| RAC875_c4465_549 | 2B | 2.26E-06 | 5.28E-06 | A/G | 699108736 | 0.90 | 0.09 | 299 | 270 | 29 |
| IACX8096 | 2B | 3.22E-06 | 5.57E-06 | G/C | 691780864 | 0.86 | 0.13 | 299 | 260 | 39 |
| RAC875_c103094_120 | 2B | 2.43E-06 | 9.05E-06 | A/C | 698303834 | 0.90 | 0.09 | 299 | 271 | 28 |
| Excalibur_c7449_587 | 2A | 2.50E-06 | 4.18E-06 | T/G | 715333875 | 0.87 | 0.12 | 299 | 262 | 37 |
| RAC875_c17455_152 | 2A | 2.93E-06 | 4.64E-07 | T/C | 717146211 | 0.90 | 0.09 | 299 | 271 | 28 |
| IACX6309 | 2A | 7.77E-06 | 4.85E-06 | A/G | 715333830 | 0.89 | 0.10 | 299 | 267 | 32 |
| RAC875_c3397_274 | 2A | 2.26E-06 | 1.20E-06 | T/G | 715333165 | 0.89 | 0.10 | 299 | 267 | 32 |

SNP: Single nucleotide polymorphism; Chr: Chromosome; Pos(bp): Physical position of marker on the wheat reference genome; MJF: Major allele frequency; MAF: Minor allele frequency; N: Total number of genotypes; AA and BB: Alternate genotypes at a specific SNP marker

| **Table S2**: Single nucleotide polymorphism markers associated with the mean of cadmium concentration in the hard-red winter wheat association panel. | | | | | | | | | |  |
| --- | --- | --- | --- | --- | --- | --- | --- | --- | --- | --- |
| SNP | Chr | P-values | Alleles | Pos(bp) | MJF | MAF | N | AA | BB | |
| Kukri_c29560_455 | 5A | 8.82E-08 | T/C | 581302317 | 0.70 | 0.29 | 299 | 211 | 88 | |
| IWA7579 | 5A | 9.47E-08 | A/G | 581108961 | 0.70 | 0.29 | 299 | 212 | 87 | |
| IWA6681 | 5A | 1.13E-07 | T/G | 580939178 | 0.70 | 0.29 | 299 | 212 | 87 | |
| IWA1752 | 5A | 1.13E-07 | A/G | 580946575 | 0.70 | 0.29 | 299 | 210 | 89 | |

SNP: Single nucleotide polymorphism; Chr: Chromosome; Pos(bp): Physical position of marker on the wheat reference genome; MJF: Major allele frequency; MAF: Minor allele frequency; N: Total number of genotypes; AA and BB: Alternate genotypes at a specific SNP marker

**Figures**

Figure S1: Principal component analysis showing the population structure in the hard winter wheat association panel. The different colors represent the sub-populations of red wheat and winter wheat.

**Figure S2**: Quantile-quantile (QQ) plot from (a) double generalized linear model and (b) hierarchical generalized linear model shown in the circular Manhattan plot. The QQ plot shows the deviation of observed p-values (blue color) from expected p-values (red line) and are sorted from largest to smallest in the plot. Note that some markers in (a) have -log_10_ (p-value) equal to zero because of the same residual variance across the allelic groups for a marker.

**Figure S3**: Linkage disequilibrium block (< 1 kb) on chromosome 2A in the vQTL region. Three markers within the 666 bp interval formed a single haplotype block.

**Figure S4**: Linkage disequilibrium blocks on chromosome 2B in the vQTL region of 691,780,716 to 699,827,018 bp. A four linkage disequilibrium blocks of less than 1,000 bp, 1 kb, 1 kb, and 204 kb within the vQTL region.

**Figure S5**: Violin plot showing the differences in the mean and variance of Cadmium concentration with alternative marker allele groups coded as AA and BB for the top three significant markers associated with mvQTL on chromosome 5A. Differences in both mean and variance can be observed across the marker groups indicating that these markers affect both mean and variance heterogeneity.
